# The relationship between oral frailty and oral dysbiosis among hospitalized patients aged older than 50 years

**DOI:** 10.1002/cre2.890

**Published:** 2024-05-30

**Authors:** Yen‐Chin Chen, En‐Ni Ku, Pei‐Fang Tsai, Che‐Wei Lin, Nai‐Ying Ko, Shun‐Te Huang, Jiun‐Ling Wang, Yi‐Ching Yang

**Affiliations:** ^1^ College of Medicine National Sun Yat‐sen University Kaohsiung Taiwan; ^2^ Department of Nursing, College of Medicine National Cheng Kung University Tainan Taiwan; ^3^ Department of Nursing Linkou Chang Gung Memorial Hospital Taipei Taiwan; ^4^ Department of Pathology, National Cheng Kung University Hospital, College of Medicine National Cheng Kung University Tainan Taiwan; ^5^ Department of Biomedical Engineering, College of Engineering National Cheng Kung University Tainan Taiwan; ^6^ Division of Pediatric Dentistry and Special Care Dentistry Kaohsiung Medical University Hospital Kaohsiung Taiwan; ^7^ Department of Medicine, College of Medicine National Cheng Kung University Tainan Taiwan; ^8^ Department of Internal Medicine, National Cheng Kung University Hospital College of Medicine, National Cheng Kung University Tainan Taiwan; ^9^ Department of Family Medicine, National Cheng Kung University Hospital, College of Medicine National Cheng Kung University Tainan Taiwan; ^10^ Department of Family Medicine, College of Medicine National Cheng Kung University Tainan Taiwan; ^11^ Department of Geriatric and Gerontology, National Cheng Kung University Hospital College of Medicine, National Cheng Kung University Tainan Taiwan

**Keywords:** hospitalized patients, middle‐age and older adults care, oral dysbiosis, oral frailty

## Abstract

**Objective:**

This study aimed to clarify the relationship between oral frailty and oral dysbiosis among hospitalized patients aged ≥ 50 years.

**Methods:**

A prospective observational study was conducted. Number of teeth, masticatory ability, articulatory oral motor skill, tongue pressure, swallowing pressure, and choking were used to assess oral frailty. Saliva samples were collected from the oral cavity for bacterial culture.

**Results:**

A total 103 in patients enrolled and 53.4% suffered from oral frailty. Oral frailty was found to have a 3.07‐fold correlation with the presence of *Enterobacterales* in the oral cavity (*p* = 0.037), especially in poor articulatory oral motor skill, which showed at greater risk of *Enterobacterales* isolated from the oral cavity by 5.58‐fold (*p* = 0.01).

**Conclusion:**

Half of hospitalized patients was found to have oral frailty that was related to more *Enterobacterales* in the oral cavity. This evidence suggests that the enhancement of articulatory oral motor skills may serve as a potential strategy for mitigating the presence of Enterobacterales within the oral cavity.

## 1 INTRODUCTION

The multifaceted nature of oral health, recognized as a key indicator of overall health and well‐being, extends beyond the mere absence of disease (Glick et al., 2016). It encompasses a spectrum of condition and functions, including disease status, physiological capacities such as speaking, smiling, chewing and swallowing, and psychological function that impact an individual's ability to engage in these activities (Glick et al., 2016). A concept of growing interest within this domain is oral frailty, which represents a novel and significant factor affecting adverse health‐related outcomes such as physical frailty, functional disability, and mortality (Dibello et al., 2023; Tanaka et al., 2018). The operational definition of oral frailty, along with the development of screening tools, has been extensively a subject of discussion. The Geriatric Oral Health Assessment Index (GOHAI) (Atchison & Dolan, 1990) and Oral Health Impact Profile (OHIP) (Locker & Slade, 1993) have been instrumental in assessing oral health status in older population. The introduction of the oral frailty concept, initially in Japan in 2013, marked a pivotal moment in understanding age‐related oral health deterioration (Watanabe et al., 2020). By 2016, the Japanese Society of Gerodontology elaborated on this concept, defining oral frailty as the second stage in a continuum that includes healthy state, oral frailty, oral hypofunction, and oral dysfunction (Minakuchi et al., 2018). The conceptual framework helps in identifying older adults experiencing decreased articulation, difficulty in chewing, and slight choking or spillage while eating, symptoms that intensify as oral function deteriorates into oral hypofunction or oral dysfunction (Minakuchi et al., 2018).

Hospitalization is one of the most stressful events for older people experiencing frailty due to restrictions on physical mobility and situations of clinical instability. [8] The prevalence of physical frailty among hospitalized older adults is reported to range from 40% to 80% depending on the different study settings and the indicators of frailty applied in the study (Andela et al., 2010; Chia‐Hui Chen et al., 2019). A systematic review including 68 observational cohort and cross‐sectional studies showed that oral health status is significantly associated with mortality, physical frailty, functional disability, hospitalization, and quality of life (Hakeem et al., 2019), especially if there are only a few remaining teeth (Dibello et al. 2021, 2023). A large‐scale survey conducted in the Japanese community by Tanaka et al. (2018) showed that six items could be identified as indicators of oral frailty. These factors include the number of natural teeth, chewing ability, articulatory oral motor skills, tongue pressure, and subjective difficulties in eating and swallowing. These factors were found to have a highly correlation with physical frailty and increased risk of mortality (Tanaka et al., 2018). Oral frailty, coupled with the progressive reduction in muscle bulk mass and strength, can lead to chewing and swallowing dysfunction (Ebihara et al., 2016). Dysfunction in oral environment can precipitate a shift toward the dominance of pathogenic bacteria, superseding commensal or beneficial bacteria, a condition indicative of dysbiosis (Costalonga & Herzberg, 2014; Maier, 2023). This oral dysbiosis potential places hospitalized older adults at an elevated risk of developing pneumonia (Omura et al., 2021; Quagliarello et al., 2005). It is postulated that bacteria present in the oral cavity may serve as potential pathogens implicated in the etiology of respiratory infections (Gomes‐Filho et al., 2010; Scannapieco, 1999). A narrative review has revealed that there is an emerging need for early identification and management of oral frailty in older adults to prevent frailty (Dent et al., 2023).

Oral frailty is highly related to worse functional recovery, prolonged time to discharge, and increased in‐hospital mortality rates (Shiraishi et al., 2019). Few studies have reported the prevalence of oral frailty among hospitalized patients, and the existing studies all came from the same study group led by Shiraisi et al. (2017, 2019, 2021) In addition, it is unclear whether this decline in oral function is related to different forms of oral dysbiosis. We aimed to investigate the prevalence of oral frailty among middle‐aged and older hospitalized patients and to identify the impact of oral frailty on oral dysbiosis in the oral cavity.

## 2 METHODS

### 2.1 Study design and setting

This prospective observational study was conducted in a large 50‐bed general medical ward in a medical center from March to December 2020. The hospital has more than 1300 beds and employs more than 5000 people and offers tertiary referral services for 1.8 million people in southern Taiwan. The general medical ward unit offers comprehensive medical care encompassing hepatobiliary and gastroenterology, infectious diseases, and cardiology services. In the general medical ward, upon admission, we regularly assess the patients' ability to carry out oral care on their own and determine the level of assistance they may require. We also emphasize the importance of oral hygiene to both patients and their families. It is recommended that oral hygiene be performed at least twice daily, specifically upon waking up in the morning and before going to bed. To ensure compliance with these recommendations, our nurses provide gentle reminders to patients and their families during medication administration or when checking vital signs.

### 2.2 Participants

Participants aged 50 or older were recruited for this study. For the purposes of this study, in patients were excluded if they had ≤3 days of hospitalization (*n* = 18), cognitive function impairment defined as a General Practitioner Assessment of Cognition [GPCOG] score less than 5 (*n* = 178), (Pirani et al., 2010) were ever diagnosed with neurological disorders (i.e., Parkinson's disease or stroke) (Morley, 2020) (*n* = 27) or were in fluctuations in vital signs and lab data or had an uncertain prognosis (*n* = 28). The data analyzed in the present study were from 103 hospitalized patients who met the inclusion criteria. The required sample size was calculated using the statistical software GPower 3.1.9.2. The coefficient of determination value was computed by inputting a value of 0.539, thereby establishing the relationship between oral health and frailty scores with an effect size of 0.539 (Genç & Uslu, 2024), a type 1 error rate of 0.05, and a sample size of 103, the power of the study was determined to be 100%.

### 2.3 Data collection procedure

This study was approved by the ethics committee (Approval No. A‐ER‐108‐397). This research was performed in accordance with relevant regulations. Data collection was carried out by two nurse professionals (Y. C. Chen and E. N. K. u.) who have successfully completed a 120‐h educational program in the assessment of oral health and oral frailty. Additionally, they have gained valuable clinical experience in special needs dentistry. To mitigate data collection bias, we conducted an interexaminer reliability assessment by completing assessments for two cases at two different time points, in the presence of a senior dentist (Dr. S. T. Huang). The agreement on the relevance of oral health and oral frailty, as determined by constructed questionnaires and a standardized protocol, was 92% and 95%, respectively.

We assessed patients' oral health, frailty, and dysbiosis upon admission and on the day of discharge, after obtaining informed consent from the participants. All participant data were anonymized to protect privacy, and personally identifiable information was removed from the data set before analysis. Confidentiality of data was maintained through encrypted data storage devices accessible only to the research team members.

### 2.4 Measurements

#### 2.4.1 Oral frailty

We modified the evaluation of oral frailty using the results of Tanaka's research (Tanaka et al., 2018). We used objective swallowing pressure measures and subjectively experienced choking within 1 month instead of subjective eating and swallowing difficulty. Our assessment consisted of the following six items for screening participants with oral frailty: (1) number of teeth: If the total number of teeth including natural teeth and fixed dentures is less than 20, it indicates a problem, (Tanaka et al., 2018) (2) masticatory ability: poor masticatory ability was defined as either reporting difficulty or inability to eat or reporting consumption of fewer than four food groups, (Hsu et al., 2012) (3) articulatory oral motor skill. The participants were instructed to repetitively produce each of the syllables/pa/,/ta/, and/ka/for a duration of 5 s each. Poor oral motor skill was defined as the pronunciation of the “Ta” sound was found to be less frequent than that of “Pa” and “Ka,” (Iwao et al., 2019; Tanaka et al., 2018) (4) tongue pressure: tongue pressure was assessed using the Iowa Oral Performance Instrument (IOPI) (Adams et al., 2013). A validity study showed IOPI was highly correlated to tongueometer (Curtis et al., 2023). A mean tongue pressure below 30.0 kPa, based on three measurements, was considered indicative of low tongue pressure, (Minakuchi et al., 2018) (5) abnormal swallowing pressure, which was defined as a tongue pressure of less than 15.46 kPa during swallowing, and (6) choking: patients self‐reported whether they had ever experienced choking. The detailed measured information has been explained in detail previously (Chen et al., 2024).

Oral frailty was determined using the methods introduced by Tanaka et al. (2018): nonoral frailty, 0 points; preoral frailty, 1–2 points; and oral frailty, ≥3 points. The oral frailty measure was performed by accredited trained oral assessment researchers (Y.‐C. Chen and E.‐N. K. u.). All oral frailty reports were verified by a board‐certified oral care specialist (S.‐T. Huang).

### 2.5 Oral health

The Oral Health Assessment Tool (OHAT) is modified from the Brief Oral Health Status Examination (BOHSE), which covers the current oral health status of patients, including contributing factors to the risk of oral disease; it also highlights the need for referral. The OHAT consists of the following eight items: lips, tongue, gums/tissues, saliva, natural teeth, dentures, oral cleanliness, and dental pain. Each item is divided into three stages: 0 = healthy, 1 = oral changes, and 2 = unhealthy. The higher the score, the worse the oral health. The reliability for OHAT categories ranges from 72.6% to 92.6% (Chalmers et al., 2005).

### 2.6 Pneumonia‐associated oral bacteria

Gargling water samples were collected from the participants for the purpose of bacterial isolation and identification. The collection took place on both the day of admission and the day of discharge, specifically during the early morning hours when participants had not consumed their meals and teeth brush. We asked the participant to gargle with 20 mL of normal saline for approximately 20 s. The standard procedure for gargling was rinsing the full mouth at least three times. We collected the gargling water in a bacterial collection bottle and sent it for bacterial culture within 2 h.

In the laboratory, gargling water was inoculated onto a blood agar plate, chocolate agar, and eosin‐methylene blue agar with a disposable 10 µL standard loop (BD Diagnostics, US/Japan), and the inoculated plates were incubated for 24 h at 35°C and 5% CO_2_ for bacterial growth. The identification of bacteria was supplemented by VITEK MS (bioMérieux S. A, 69280 Marcy l’Étoile, France) and the results of the quantitative culture are shown in colony forming units per milliliter. The inoculated plates were incubated for more than 24 h to isolate pneumonia‐associated oral bacteria.

### 2.7 Covariates

Medical records data were collected from the electronic medical system for the following items: length of hospitalization in days, age, sex, level of education, major diagnosis at admission, number of comorbidities, primary caregiver, and history of admission to the intensive care unit (ICU), if any.

### 2.8 Statistical analysis

Comparisons of the differences in oral frailty types were performed using the *χ* ^2^ test with Fisher's exact test (for dichotomous variables) and one‐way analysis of variance (ANOVA, for continuous variables). A paired *t* test and McNemar's test were used to assess the differences in oral frailty between the time of admission and discharge. The Kruskal–Wallis test was used to analyze the mean differences of eight subscales of oral health and the distribution of oral bacteria counts among the three groups. Finally, we assessed the relationships between oral dysbiosis and oral frailty using multiple logistic regression after controlling for covariates. A *p* < 0.05 was considered statistically significant. The analysis was performed with SPSS v.22.2 (IBM Corp.).

## 3 RESULTS

### 3.1 Demographic data

Out of a total of 548 individuals screened between May and December 2020, 251 were excluded due to various reasons. Consequently, 297 middle‐aged and older hospitalized patients were deemed eligible for the study, of which 103 accept and successfully completed the protocol (Figure S1).

A total of 103 patients, with a mean age at study enrollment of 65.92 ± 11.77 years, were classified into three groups based on the oral frailty assessment: 6.8% in the nonfrailty group, 9.8% in the prefrailty group, and 53.4% in the frailty group. The main diagnoses among the hospitalized patients were infectious diseases (35.9%), gastrointestinal diseases (32.0%), and others (32.0%). The average length of stay in our cohort was 7.45 ± 5.03 days. Compared with hospitalized patients without oral frailty or prefrailty, those with oral frailty tended to be older and were more likely to be diagnosed with gastrointestinal disease (Table 1).

**Table 1:** Characteristic data among hospitalized patients (*N* = 103).

Variables	Overall		Oral frailty						*p* value
			Nonfrailty (*n* = 7, 6.8%)		Prefrailty (*n* = 41, 39.8%)		Frailty (*n* = 55, 53.4%)		
	*n*	%	*n*	%	*n*	%	*n*	%	
Hospitalized days, mean (SD)	7.5 (5.0)		7.7 (7.0)		7.5 (4.8)		7.4 (5.0)		0.52
Age, mean (SD)	65.9 (11.8)		62.1 (8.1)		61.95 (10.7)		69.36 (12.0)		<0.01
Gender									0.79
Male	61	59.2	5	71.4	24	58.5	32	58.2	
Female	42	40.8	2	28.6	17	41.5	23	41.8	
Level of education									0.23
Under elementary school	59	57.3	2	28.6	23	56.1	34	61.8	
Between junior and high school	38	36.9	5	71.4	14	34.1	19	34.5	
University degree or above	6	5.8	0	0.0	4	9.8	2	3.6	
Number of comorbidities	1.6, 1.2		1.1, 0.7		1.5, 1.3		1.8, 1.1		0.31
Comorbidities									
Hypertension	53 (51.3)		3 (42.9)		20 (48.8)		30 (56.6)		0.77
Type 2 diabetes	38 (36.9)		5 (71.4)		14 (34.1)		19 (34.5)		0.15
COPD	11 (10.7)		0 (0.0)		3 (7.3)		8 (14.5)		0.34
CVDs	20 (19.4)		0 (0.0)		5 (12.2)		15 (27.3)		0.07
Cancer	16 (15.5)		0 (0.0)		6 (14.6)		10 (18.2)		0.45
Hepatitis B, C	1 (1.0)		0 (0.0)		0 (0.0)		1 (1.0)		0.64
Liver cirrhosis	6 (5.8)		0 (0.0)		2 (4.9)		4 (7.3)		0.70
CRF	11 (10.7)		0 (0.0)		6 (14.6)		5 (9.1)		0.44
Immunodeficiency	0 (0.0)		2 (4.9)		4 (7.3)		0 (0.0)		0.70
Main admitting diagnosis									0.01
Infectious diseases	37 (35.9)		3 (42.9)		20 (48.8)		14 (25.5)		
GI diseases	33 (32.0)		0 (0.0)		7 (17.1)		26 (47.3)		
Others	33 (32.0)		4 (57.1)		14 (34.1)		15 (27.3)		
Use of antibiotics during the study period	76 (73.8)		5 (71.4)		31 (75.6)		40 (72.7)		0.94
Main Caregiver									0.79
None	9	8.7	1	14.3	3	7.3	5	9.1	
Family	84	81.6	6	85.7	35	85.4	43	78.2	
Local assistance	6	5.8	0	0.0	1	2.4	5	9.1	
Foreign assistance	4	3.9	0	0.0	2	4.9	2	3.6	
Ever admitted to ICU	20	19.4	1	14.3	4	9.8	15	27.3	0.09

Abbreviations: COPD, chronic obstructive pulmonary disease; CVDs, cerebrovascular diseases; CRF, chronic renal failure; GI, gastrointestinal; ICU, intensive care unit; SD, standard deviation.

### 3.2 Changes in oral frailty between admission and discharge among hospital patients

We evaluated the changes in oral frailty status between admission and discharge. The results showed an improvement in oral frailty at discharge (53.4% vs. 38.8%, *p* < 0.001), especially in mean tongue pressure. The mean tongue pressure significantly increased after acute illness recovery (mean kPa, 29.5 vs. 33.6, *p* < 0.01) (Table 2).

**Table 2:** Differences in oral frailty between admission and discharge (*N* = 103).

	*n* (%)		
Items	Admission	Discharge	*p* Value
Less than 20 normal teeth	40 (38.8)	40 (38.8)	1.00
Poor masticatory ability	35 (34.0)	34 (33.0)	0.10
Difficulty making a “ta” sound	46 (44.7)	36 (35.0)	0.47
Weak tongue pressure (kPa)	53 (51.5)	37 (35.9)	0.25
Mean (SD)	29.5 (15.0)	33.6 (15.8)	**<0.01**
Weak swallowing pressure (kPa)	62 (60.2)	45 (43.7)	1.00
Mean (SD)	19.8 (10.6)	21.2 (11.2)	0.39
Choking	18 (17.5)	18 (17.5)	0.10
Overall (scores ≥ 3)	55 (53.4)	40 (38.8)	**<0.001**

*Note*: Bold font indicates statistical significance.

Abbreviation: SD, standard deviation.

### 3.3 The differences in the eight subscales of the OHAT among the three groups

The oral frailty group had significantly worse oral health than the nonfrailty and prefrailty groups (mean OHAT scores, 3.8 vs. 3.0 vs. 2.7, *p* = 0.01). For the eight subscales of the OHAT among the three groups, we found a significant difference with respect to the number of natural teeth (*p* = 0.04) (Table 3).

**Table 3:** Differences in the eight subscales of the OHAT among the three groups.

Variables	Nonfrailty		Prefrailty		Frailty		*p* Value
	Mean	SD	Mean	SD	Mean	SD	
Total scores of OHAT at baseline	3.0	2.0	2.7	1.7	3.8	1.7	**0.01**
Lips	0.1	0.3	0.1	0.3	0.2	0.4	0.87
Tongue	0.3	0.5	0.4	0.5	0.6	0.5	0.21
Gums and tissues	0.2	0.4	0.2	0.4	0.1	0.3	0.49
Saliva	0.2	0.4	0.3	0.5	0.4	0.6	0.56
Natural teeth	0.6	0.5	0.7	0.8	1.1	0.9	**0.04**
Dentures	0.1	0.3	0.1	0.5	0.3	0.7	0.31
Oral cleanliness	0.8	0.7	1.0	0.8	1.1	0.8	0.58
Dental pain	0.0	0.0	0.0	0.0	0.7	0.3	0.17

*Note*: Bold font indicates statistical significance.

Abbreviations: OHAT, oral health assessment tool; SD, standard deviation.

### 3.4 The distribution of oral bacteria among the three groups

**Figure 1:**
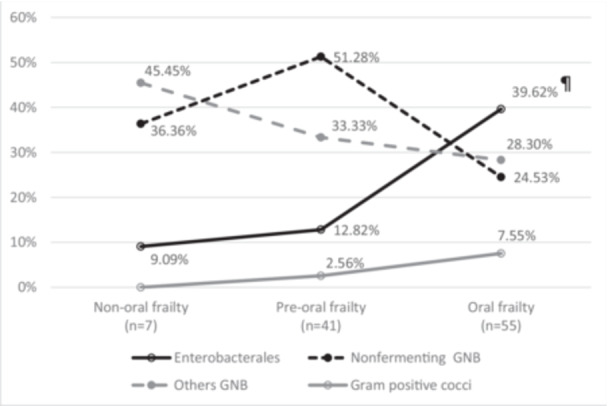
Distribution of oral bacteria counts among the three groups by four microbes. GNB, gram‐negative bacilli. ^¶^Indicates statistical significance.

There was a significant difference in the *Enterobacterales* counts among the three groups. The frailty group was associated with more *Enterobacterales* in the oral cavity than the nonfrailty and preoral frailty groups (39.62% vs. 9.09% vs. 12.82%, *p* = 0.044) (Figure 1). Considering each *Enterobacterales* in detail, the frailty group was more likely to have a higher percentage of pneumonia‐associated bacteria, such as *Klebsiella pneumoniae* (20.75%), followed by *Escherichia coli* (5.66%) and *Serratia marcescens* (5.66%), than the other two groups (Table S1).

### 3.5 Relationships between oral frailty and *Enterobacterales* in the oral cavity

In multivariate analysis in Model 1 showed that oral frailty at baseline (AOR: 3.11, 95% CI: 1.04−9.25, *p* = 0.004) correlated with a higher level of *Enterobacterales* in the oral cavity. Furthermore, we explored which specific oral measures were related to *Enterobacterales* in the oral cavity. Multivariate analysis in Model 2 showed that difficulty making the “ta” sound increased the risk of *Enterobacterales* isolation from the oral cavity by 5.58‐fold (OR = 5.58, 95% CI: = 1.46−21.40, *p* = 0.01) after adjusting for fewer than 20 normal teeth, poor masticatory ability, weak tongue and swallowing pressure, and choking (Table 4).

**Table 4:** Relationships between *Enterobacterales* detected in the oral cavity and oral frailty among middle‐aged and elderly hospitalized patients.

Variables	Univariate				Multivariate			
	OR	95% CI		*p* Value	AOR	95% CI		*p* Value
*Model 1*								
Age	1.03	0.98	1.07	0.22	1.03	0.98	1.08	0.26
Sex								
Female	Reference				Reference			
Male	0.87	0.34	2.21	0.77	1.09	0.38	3.16	0.88
Hospitalized days	1.05	0.96	1.14	0.27	1.08	0.97	1.19	0.15
Number of comorbidities	0.70	0.44	1.09	0.11	0.65	0.33	1.03	0.11
GI disease as the main diagnosis	1.50	0.57	3.93	0.41	0.95	0.32	2.86	0.93
Ever admitted to ICU	1.20	0.39	3.76	0.75	0.90	0.23	3.52	0.88
OHAT at baseline	1.11	0.87	1.42	0.39	1.07	0.79	1.46	0.65
Oral frailty at baseline	3.06	1.18	7.94	0.02	3.11	1.04	9.25	0.04
*Model 2*								
Less than 20 normal teeth	1.14	0.38	3.39	0.82	1.14	0.25	5.13	0.87
Poor masticatory ability	1.27	0.43	3.71	0.67	0.82	0.17	3.86	0.80
Difficulty making a “ta” sound	5.65	1.65	19.4	0.01	5.58	1.46	21.41	0.01
Weak tongue pressure (kPa)	1.00	0.96	1.03	0.78	0.99	0.94	1.04	0.66
Weak swallowing pressure (kPa)	0.98	0.93	1.03	0.41	0.97	0.90	1.05	0.45
Choking	1.60	0.48	5.37	0.44	1.89	0.44	8.17	0.40

Abbreviations: AOR, adjusted odds ratio; GI, gastrointestinal; ICU, intensive care unit; OHAT, Oral Health Assessment Tool; OR, odds ratio;.

## 4 DISCUSSION

Oral frailty among hospitalized patients over the age of 50 years was detected in 55 (53.4%) patients upon admission. We found that the prevalence of oral frailty in our study is lower than that in previous studies by Shiraishi Ai et al. who reported that hospitalized patients with postacute stroke or hospitalized patients with new stroke had slight to severe oral problems, 82.8% (Shiraisi et al., 2021) and 69.2% (Shiraishi et al., 2018), respectively. Some possible explanations for this inconsistency are that different oral frailty measurement tools were used and that the studies assessed different characteristics of the hospitalized patients. Previous studies have placed a larger focus on oral frailty in stroke patients. Stroke is a neurological disorder that affects the process of swallowing functions through brain damage (González‐Fernández et al., 2013). The prevalence of oral frailty among hospitalized stroke patients would be expected to be higher than that among general hospitalized patients. More large‐scale studies are needed to better understand the prevalence of oral frailty among general hospitalized patients.

This cohort evaluated oral frailty changes during hospitalization and found that the oral frailty score improved at the time of discharge. We found that oral frailty could be improved by 14.6% among hospitalized patients after recovery from an acute phase illness, particularly in terms of tongue pressure. Potential factors contributing to the observed improvement in oral frailty can be posited. Initially, hospitalization is a phase during which individuals are in a vulnerable state, experiencing heightened physical decline due to constraints imposed by bed rest and unstable physical conditions, eventually leading to a decline in muscular strength (Rosa et al., 2018). Several studies have indicated that tongue pressure is highly correlated with skeletal muscle mass index and handgrip strength (Chen et al., 2021; Sugiya et al., 2022), and tongue pressure might be more vulnerable to improvement when physiological recovery occurs among hospitalized patients. Furthermore, requiring hospitalization for more than 3 days means that patients were in a severe situation. As such, neurological integrity might be compromised upon admission, in accordance with cognitive frailty models, leading to transient weaknesses, such as physical and oral frailty (Panza et al., 2018). Some associated factors might facilitate this improvement, including the implementation of rigorous oral hygiene assessment and education protocols within the hospital environment. These protocols heighten the awareness of patients and their families toward the prevention of physical frailty (Irie et al., 2024; Liu et al., 2023). Moreover, a multidisciplinary approach that encompasses the expertize of nurses, physicians, dentists, and other healthcare professionals is integral to this process. This cooperative strategy ensures oral health is given due attention and also facilitates the provision of appropriate nutritional support and sufficient hydration (Irie et al., 2024). Frailty is manageable and reversible in hospitals, with physiotherapy (Braun et al., 2019) and nutrition therapy (Shimazaki et al., 2020) to avoid falls (Öztürk et al., 2017) and clinical deterioration (So et al., 2018) and decrease the length of hospital stay (Nguyen et al., 2019). Effective strategies for improving oral frailty among hospitalized middle‐aged and older adult patients are needed.

A qualitative study showed that due to the negative correlations between frailty and oral health, it would be more difficult to carry out adequate oral hygiene activities (Niesten et al., 2013). We found similar evidence in an epidemiological survey of 14 nursing homes indicating that bacterial count is significantly associated with poor oral health (Tohara et al., 2017). A correlation study found that *K. pneumoniae* and *E. coli* were detected in 22% of patients with acute stroke and suspected dysphagia during hospitalization. However, these bacteria were not found in healthy mouths (Perry et al., 2020).

Oral frailty was significantly correlated with *Enterobacterales* in the oral cavity. Oral frailty contributes to weak orofacial or oropharyngeal muscles, and *Enterobacterales* are indigenous gastrointestinal tract flora (Donskey, 2004). When patients have trouble swallowing, food or liquids retained in the pharynx may lead to microbial exchange between the oropharyngeal and gastric flora (Duvallet et al., 2019). Serial physiological changes may be a partial explanation for our observation. According to limited knowledge, there is no direct evidence similar to ours. This suggests that the relationship between oral frailty and oral microbial exchange remains unclear. Further studies are needed to explore the mechanism of *Enterobacterales* retention in the oral cavity of patients with oral frailty.

Our findings further revealed that the articulatory oral motor skill (difficulty in making the “ta” sound) was associated with *Enterobacterales* in the oral cavity. The probable reason could be that reflux of *Enterobacterales* occurs from the gastrointestinal tract to the oropharynx in patients with impaired articulatory oral motor skills. Older adults with oropharyngeal dysphagia are at high risk for developing aspiration pneumonia (Ortega et al., 2014). A previous study showed that colonization by respiratory pathogens was high in patients with oropharyngeal dysphagia; however, the detailed distribution of pathogens was not shown in that study (Ortega et al., 2015). Enterobacter species are a common cause of nosocomial respiratory infections; thus, further research is needed to investigate the causal relationship between *Enterobacterales* and oral frailty among hospitalized patients. Additionally, it should be determined whether improving oral frailty, including articulatory oral motor skills, could decrease oral *Enterobacterales* colonization and the risk of aspiration pneumonia.

### 4.1 Limitations

There are some limitations of our study. First, our study was not a longitudinal follow‐up, which makes it difficult to detect the long‐term correlation of oral frailty with oral *Enterobacterales*. Second, our results have limited generalizability to the older population because we only included hospitalized patients and contained few numbers of participants exhibiting non‐frailty. In addition, ascertainment of the relationships between oral frailty and oral dysbiosis was based on the observation of in patients, and it is difficult to control complex conditions during hospitalization, such as the main admitting diagnoses, antibiotic use, or disease progression. A previous animal study revealed that the effects of short‐term antibiotic therapy did not result in significant alterations in the rate of oral microbiota (Wu et al., 2020). Finally, considering that the time since the onset of the main reason for hospitalization was different, and tongue pressure is not a valid proxy measure for sarcopenia, our finding of tongue pressure improvement after acute illness control might be influenced.

## 5 CONCLUSIONS

Oral frailty is highly prevalent among hospitalized people aged over 50 years. After the acute illness was controlled, oral frailty improved, especially with regard to tongue pressure. Oral frailty may cause a disadvantageous condition, such as increased *Enterobacterales* oral colonization among middle‐aged and older adult patients. Further longitudinal research on the effect of *Enterobacterales* colonization after improvements in oral frailty status and articulatory oral motor skills is necessary.

## 6 IMPLICATIONS FOR BEHAVIORAL HEALTH

Oral frailty represents a prevalent age‐related syndrome observed among middle‐aged and older adults during hospitalization. Our study confirms a noteworthy elevation of *Enterobacterales* colonization in the oral cavity within the oral frailty group compared to both the non‐frail and pre‐frail groups. Furthermore, our findings indicate that a deficiency in articulatory oral motor skills is associated with a 5.58‐fold higher risk of Enterobacterales isolation in the oral cavity. This evidence suggests that the enhancement of articulatory oral motor skills may serve as a potential strategy for mitigating the presence of Enterobacterales within the oral cavity.

## AUTHOR CONTRIBUTIONS

**Yen‐Chin Chen, Pei‐Fang Tsai, Che‐Wei Lin, Nai‐Ying Ko, Shun‐Te Huang, Yi‐Ching Yang, Jiun‐Ling Wang**: Made substantial contributions to conception and design, or acquisition of data, or analysis and interpretation of data. **Yen‐Chin Chen, En‐Ni Ku, Pei‐Fang Tsai, Jiun‐Ling Wang**: Involved in drafting the manuscript or revising it critically for important intellectual content. **Yen‐Chin Chen, En‐Ni Ku, Pei‐Fang Tsai, Che‐Wei Lin, Nai‐Ying Ko, Shun‐Te Huang, Yi‐Ching Yang, Jiun‐Ling Wang**: Given final approval of the version to be published. Each author should have participated sufficiently in the work to take public responsibility for appropriate portions of the content. **Yen‐Chin Chen, En‐Ni Ku, Pei‐Fang Tsai, Che‐Wei Lin, Nai‐Ying Ko, Shun‐Te Huang, Yi‐Ching Yang, Jiun‐Ling Wang**: Agreed to be accountable for all aspects of the work in ensuring that questions related to the accuracy or integrity of any part of the work are appropriately investigated and resolved.

## CONFLICT OF INTEREST STATEMENT

The author declare no conflict of interest.

## ETHICS STATEMENT

This study was approved by the ethics committee (Approval No. A‐ER‐108‐397). This research was performed in accordance with relevant regulations. We assessed patients' oral health, frailty, and dysbiosis on admission and the discharge day after obtaining informed consent from the participants.

## Supporting information

Supporting information.

Supporting information.

## Data Availability

The data that support the findings of this study are available from the corresponding author, Yen‐Chin Chen, upon reasonable request.
