# Supplementary material for: The relationship between oral frailty and oral dysbiosis among hospitalized patients aged older than 50 years
Source: Clin Exp Dent Res. 2024 May 30;10(3):e890. doi: 10.1002/cre2.890 (PMC11139674; doi:10.1002/cre2.890)
Supplement: Supplementary file 1 — Supporting information. [file CRE2-10-e890-s002.docx]

Supplementary Figure 1. Recruitment process flowchart

**194** excluded due to:
 225 refused after introduction by

General practitioners

**251** excluded due to:
 18 had ≤ 3 days of hospitalization
 178 cognitive impairment
 27 ever diagnosed with neurological

disorders

28 unstable conditions or an

uncertain prognosis

**103** Completed

**297** eligible

**548** Participants aged 50 or older assessed for eligibility
