# Supplementary material for: The relationship between oral frailty and oral dysbiosis among hospitalized patients aged older than 50 years
Source: Clin Exp Dent Res. 2024 May 30;10(3):e890. doi: 10.1002/cre2.890 (PMC11139674; doi:10.1002/cre2.890)
Supplement: Supplementary file 2 — Supporting information. [file CRE2-10-e890-s001.docx]

Supplementary Table 1. Counts of oral bacteria among the three groups by four microbes (N= 103)

| Oral bacteria | Bacterial Counts | Non-oral frailty  (n=7) | | Pre-oral frailty (n=41) | | Oral frailty  (n=55) | |
| --- | --- | --- | --- | --- | --- | --- | --- |
|  | Mean (SD) | n | % | n | % | n | % |
| ***Gram-positive cocci*** | 31200.00 (302.91) |  |  |  |  |  |  |
| *Staphylococcus aureus* | 30200.00 (293.20) | NA | NA | 1 | 2.56 | 3 | 5.66 |
| *Rothia mucilaginosa* | 1000.00 (9.71) | NA | NA | NA | NA | 1 | 1.89 |
| ***Enterobacterales*** | 83000.00 (805.83) |  |  |  |  |  |  |
| *Citrobacter freundii* | 10000.00 (97.09) | NA | NA | 1 | 2.56 | NA | NA |
| *Enterobacter cloacae complex* | 800.00 (7.77) | NA | NA | NA | NA | 2 | 3.77 |
| *Escherichia coli* | 30200.00 (293.20) | NA | NA | 1 | 2.56 | 3 | 5.66 |
| *Klebsiella aerogenes* | 500.00 (4.85) | NA | NA | NA | NA | 1 | 1.89 |
| *Klebsiella pneumoniae* | 32900.00 (319.42) | 1 | 9.09% | 3 | 7.69 | 11 | 20.75 |
| *Proteus mirabilis* | 100.00 (0.97) | NA | NA | NA | NA | 1 | 1.89 |
| *Serratia marcescens* | 8500.00 (82.52) | NA | NA | NA | NA | 3 | 5.66 |
| ***Nonfermenting GNB*** | 30000.00 (1036.89) |  |  |  |  |  |  |
| *Acinetobacter* | 38100.00 (369.90) | 2 | 18.18 | 8 | 20.51 | 4 | 7.55 |
| *Cupriavidus gilardii* | 300.00 (2.91) | NA | NA | NA | NA | 1 | 1.89 |
| *Chryseobacterium* | 13400.00 (130.10) | NA | NA | 3 | 7.69 | 2 | 3.77 |
| *Elizabethkingia anophelis* | 900.00 (8.74) | 1 | 9.09 | NA | NA | NA | NA |
| *Ralstonia mannitolilytica* | 1200.00 (11.65) | NA | NA | NA | NA | 1 | 1.89 |
| *Stenotrophomonas maltophilia* | 11000.00 (106.80) | NA | NA | 4 | 10.26 | 2 | 3.77 |
| *Pseudomonas aeruginosa* | 41900.00 (406.80) | 1 | 9.09 | 5 | 12.82 | 3 | 5.66 |
| ***Other GNB*** | 186600.00 (1811.65) |  |  |  |  |  |  |
| *Capnocytophaga* | 113300.00 (1100.00) | 4 | 36.36 | 5 | 12.82 | 11 | 20.75 |
| *Haemophilus* | 31200.00 (302.91) | 1 | 9.09 | 5 | 12.82 | 1 | 1.89 |
| *Unidentified GNB* | 42100.00 (408.74) | NA | NA | 3 | 7.69 | 3 | 5.66 |

Note: GNB, gram-negative bacilli, NA, non-available
